# The combination of nano-calcium sulfate/platelet rich plasma gel scaffold with BMP2 gene-modified mesenchymal stem cells promotes bone regeneration in rat critical-sized calvarial defects

**DOI:** 10.1186/s13287-017-0574-6

**Published:** 2017-05-25

**Authors:** Zunpeng Liu, Xue Yuan, Gabriela Fernandes, Rosemary Dziak, Ciprian N. Ionita, Chunyi Li, Changdong Wang, Shuying Yang

**Affiliations:** 1 Department of Oral Biology, School of Dental Medicine, University of Buffalo, The State University of New York, Buffalo, NY USA; 2 Developmental Genomics Group, New York State Center of Excellence in Bioinformatics and Life Sciences, University of Buffalo, The State University of New York, Buffalo, NY USA; 3 Department of Anatomy and Cell Biology, School of Dental Medicine, University of Pennsylvania, Philadelphia, PA 19104 USA; 4 Department of Orthopedics, Fourth Affiliated Hospital, China Medical University, Shenyang, China; 5 Departments of Biomedical Engineering and Neurosurgery, Toshiba Stroke And Vascular Research Center, University of Buffalo, The State University of New York, Buffalo, NY USA

**Keywords:** Bone morphogenetic protein 2, Mesenchymal stem cells, Platelet-rich plasma, Critical-sized bone defect, Bone tissue engineering, Bone scaffolds

## Abstract

**Background:**

Mesenchymal stem cells (MSCs) can be differentiated into an osteoblastic lineage in the presence of growth factors (GFs). Platelet-rich plasma (PRP), which can be easily isolated from whole blood, contains a large amount of GFs, and, therefore, promotes bone growth and regeneration. The main goal of this work was to develop and investigate the effect of a new sandwich-like bone scaffold which combines a nano-calcium sulfate (nCS) disc along with PRP fibrin gel (nCS/PRP) with BMP2-modified MSCs on bone repair and regeneration in rat critical-sized calvarial defects.

**Methods:**

We evaluated the cytotoxicity, osteogenic differentiation and mineralization effect of PRP extract on BMP2-modified MSCs and constructed a sandwich-like nCS/PRP scaffold (mimicking the nano-calcium matrix of bone and carrying multi GFs in the PRP) containing BMP2-modified MSCs. The capacity of this multifunctional bone regeneration system in promoting bone repair was assessed in vivo in a rat critical-sized (8 mm) calvarial bone defect model.

**Results:**

We developed an optimized nCS/PRP sandwich-like scaffold. Scanning electron microscopy (SEM) results showed that nCS/PRP are polyporous with an average pore diameter of 70–80 μm and the cells can survive in the nCS/PRP scaffold. PRP extract dramatically stimulated proliferation and differentiation of BMP2-modified MSCs in vitro. Our in vivo results showed that the combination of BMP2-modified MSCs and nCS/PRP scaffold dramatically increased new bone regeneration compared with the groups without PRP and/or BMP2.

**Conclusions:**

nCS/PRP scaffolds containing BMP2-modified MSCs successfully promotes bone regeneration in critical-sized bone defects. This system could ultimately enable clinicians to better reconstruct the craniofacial bone and avoid donor site morbidity for critical-sized bone defects.

**Electronic supplementary material:**

The online version of this article (doi:10.1186/s13287-017-0574-6) contains supplementary material, which is available to authorized users.

## Background

For critical-sized bone defects, conventional treatment methods use autografts, allografts, xenografts, and synthetic bone grafts. Although these grafts have shown satisfactory results of bone regeneration, they often possess disadvantages [1] including donor site morbidity, shortage of autografts, risk of disease transmission and immune rejection [2, 3]. These disadvantages potentiate a need for developing newer materials as bone grafts that can demonstrate better results, reduce costs, and overcome problems associated with existing grafts.

It has been well known that stem cells, especially mesenchymal stem cells (MSCs), have been used for hard tissue engineering over the years [4–6]. The advantages in the use of MSCs resides in the fact that unlike autogenous grafts, which are not easily available, even a small portion of MSCs derived from tissue can be expanded in in vitro culture for transplantation into defects to advance repair and remodeling of several tissues [7, 8]. Since these MSCs can be autologous (obtained from the same patient), this decreases the risk of disease transmission and immune rejection.

In order to promote MSC differentiation and proliferation, it is essential to have the presence of a catalyst, which can accelerate this potential without affecting its cellular structure and biology [9], and is inexpensive, biocompatible and osteoconductive [10, 11]. Bone morphogenetic proteins (BMPs) have been examined for their potential to promote osteoblast proliferation and maturation [12], and are extensively used in bone regeneration [13]. Our previous studies have proved that BMP2 in combination with MSCs is able to repair critical-sized bone defects [14, 15]. Furthermore, BMP2 genetically engineered MSCs have unique advantages for promoting bone regeneration because BMP2 expression is relatively stable, and this treatment is much less expensive than the administration of recombinant BMP2 [14].

Platelet-rich plasma (PRP) consists of several growth factors and the predominate ones are platelet-derived growth factor (PDGF), insulin-like growth factor (IGF), and transforming growth factor beta (TGF-beta) along with their isoforms [16]. PRP also consists of BMP2, which is a powerful stimulator for osteogenic differentiation of MSCs. Upon activation of PRP, these growth factors are released; however, the life span of these growth factors is only 24 hours, which makes it imperative to deliver these growth factors via an agent in order to prolong its function in bone regeneration [17–20].

Recently, nano-calcium sulfate (nCS) has been employed as a scaffold in bone regeneration. It is of enhanced physical properties, such as high surface area for growth factor adsorption, with the potential for controlling the rate of release of the adsorbed material, as well as superior mechanical strength for optimal osteoconductivity and resistance to fractures [21]. Therefore, in this study, we hypothesized that nCS provides good support for PRP, and combined them with BMP2-modified MSCs, and greatly promotes bone formation and regeneration. We found PRP extract significantly promoted the proliferation and osteogenic differentiation of MSCs in vitro. When PRP is combined with nCS and BMP2-modified MSCs, it greatly promotes bone formation in a rat critical-sized (8 mm) calvarial bone defect model.

## Methods

### PRP preparation

PRP and PRP gel were prepared by a modification of the method of Landesberg et al. [22] and have been previously described [23]. Briefly, Sprague-Dawley (SD) rats at 8-week old were anesthetized with tribromoethanol (Avertin, intraperitoneal injection) and the whole blood was collected via retro-orbital puncture. The blood was anticoagulated with 3% acid-citrate dextrose (1/10 volume) and centrifuged at 200 × g at 4 °C for 10 min, then the upper plasma and intermediate layer with fewer red blood cells were collected into a new tube. This new tube was centrifuged again at 200 × g for 10 min at 4 °C. The upper layer is called platelet-poor plasma (PPP). Most PPP was removed, and only 0.35 ml PPP was used to resuspend PRP in the bottom. Thrombin (50 U per 1 ml PRP) was added to release the factors from the concentrated platelets and yield PRP gel, which were used for our in vivo experiment (Additional file 1: Figure S1A). PRP gel was centrifuged at 1500 × g for 5 min, and then the upper solution was harvested as PRP extract. PRP extract contains abundant growth factors and was used immediately after its preparation for our in vitro experiment (Additional file 1: Figure S1A).

### Osteogenic differentiation and BMP2 gene transfer

Rat MSCs were derived from the bone marrow of 6–8-week-old SD rats and identified as we described previously [14].

BMP2 adenovirus was produced and titer tested as previously described [14]. Ad-GFP (as a control) or Ad-BMP2 adenovirus (MOI = 100) was added to MSCs in serum-free medium. After 4 h, serum was added to a final concentration of 2% for overnight culture. Cells were then switched to osteogenic media containing 50 μg/mL of ascorbic acid, 10 mM β-glycerophosphate and 10^-8^ M dexamethasone.

### Cell proliferation and viability

MSCs were treated with different concentration of PRP extract (2.5, 5, and 10%) for 7 days. At day 1, day 3 and day 7, cell density was measured by using CellTiter 96 AQueous One Solution Cell Proliferation Assay kit (Promega, Madison, WI, USA). Optical density values were measured at 490 nm using a microplate reader. Three independent biological replicates were assessed.

### Alkaline phosphatase (ALP) assay and Alizarin Red staining

MSCs were induced with osteogenic medium for 7 days for ALP assay and 16 days for Alizarin Red staining. For ALP activity assay, cells were harvested, and the supernatants were used for ALP activity assay as described previously [14]. For Alizarin Red staining, cells were stained with 40 mM of Alizarin Red S solution (pH 4.1–4.4). 10% cetylpyridinium chloride (in 10 mM sodium phosphate, pH 7.0) was used to destrain the cells and the optical density (OD) was measured at 562 nm by an AD 340 microplate reader. Three independent biological replicates were assessed.

### Scaffold preparation

nCS discs were made according to the method of Park et al. [24]. Three-dimensional printed molds (1 mm thickness × 7 mm diameter) were used to construct the nCS discs. The nCS discs were formulated with nCS and alginate. Alginate was dissolved in PBS to prepare a 10% solution (w/v), and then the pH was adjusted to 7.4–7.6. nCS powder (135 mg) was mixed with alginate solution (150 μl). The total mass of nCS and alginate was 150 mg. After 30 min following the mixing and filling into the 3D molds with nCS/alginate, the solid nCS discs were formed. Thereafter, the surface of the discs was spread with 40 μl PRP, and then 4 μl calcium chloride (10%) was used to convert the PRP to a gel as well as activate the growth factors (Additional file 1: Figure S1B). The PRP gel (1 mm thick) attached to nCS disc to form an nCS/PRP scaffold.

### Scanning electron microscopy

Rat MSCs were seeded on the surface of each nCS/PRP scaffold and cultured for 24 h. Then, the samples were rinsed in 0.1 M phosphate buffer twice and then left in 0.1 M sodium cacodylate (Sigma-Aldrich, St. Louis, MO, USA) for 30 min. The samples were then dehydrated in gradient ethanol and treated with hexamethyldisilizane. The specimens were sputter coated with carbon and observed under a scanning electron microscope.

### Rat critical-sized calvarial bone defect model

The protocol was reviewed and approved by the University at Buffalo Animal Care and Use Committee. Forty-two male SD rats at 8-week old were used in this study. The animals were divided into seven groups: group 1, blank control (defect only); group 2, nCS only; group 3, nCS + PRP; group 4, nCS (MSCs), group 5, nCS (MSCs) + PRP, group 6, nCS (MSCs/B2) and group 7, nCS (MSCs/B2) + PRP. The scaffold preparation was showed in Additional file 1: Figure S1B. The surgery process for generation of rat critical-sized calvarial bone defect model and scaffold transplantation were performed as shown in Additional file 1: Figure S1C [14]. Briefly, rats were anesthetized with isoflurane/O_2_ gas. The scalps covering the calvarial vault were shaved. An incision was made along the midline of each rat. An 8-mm-diameter trephine bur was used to drill a standardized, round, segmental defect around the sagittal suture. Scaffolds were placed into the defects, and periosteum (pericranium) and skin were sutured. Buprenorphine (0.05 mg/kg), 2% lidocaine (20 mg/ml) and carprofen (5 mg/kg) were given before, during, and after surgery. Eight weeks later, the animals were euthanized using CO_2_, and calvarial bones were harvested for further analysis.

### Analysis of in vivo bone formation

Bone density measurements (BMD, g/cm^2^) of all implants from the rats were performed using a LUNAR PIXImus bone densitometer (Lunar Corp., Madison, WI, USA). LUNAR PIXImus software was used to analyze the scanned data. On the computerized scan plots, five regions of interest per implant were selected to measure the BMD of the defect area, and the average values were taken as the final result per mouse. This allowed BMD measurement in close relation to the bone regeneration area by the software excluding pixels.

For histological analysis, half of the calvarial bones were decalcified and cut into 5 μm sections. The sections were then stained with hematoxylin and eosin. The ratio of the bone area in the implants against the total implant tissue of section was quantified using NIH Image J software and defined as the percentage of bone volume in the implant/total volume of the implant.

### Micro-CT

The harvested implants were fixed and scanned using a custom-build micro-CT system (n = 4 for each group). The data were analyzed with MicroView 3D Image Viewer & Analysis Tool (GE Medical Systems, London, ON, Canada).

### Statistical analysis

Statistical analysis was performed using SPSS software version 17.0 (SPSS Inc., Chicago, IL, USA). Experimental data were reported as mean ± standard deviation of triplicate independent samples. Data were analyzed using Student’s *t* test and one-way analysis of variance, and Tukey’s HSD test was applied as a post hoc test if statistical significance was determined. A value of *p* ≤ 0.05 was considered statistically significant.

## Results

### PRP promotes BMP2-modified MSCs proliferation and osteogenic differentiation in vitro

**Fig. 1:**
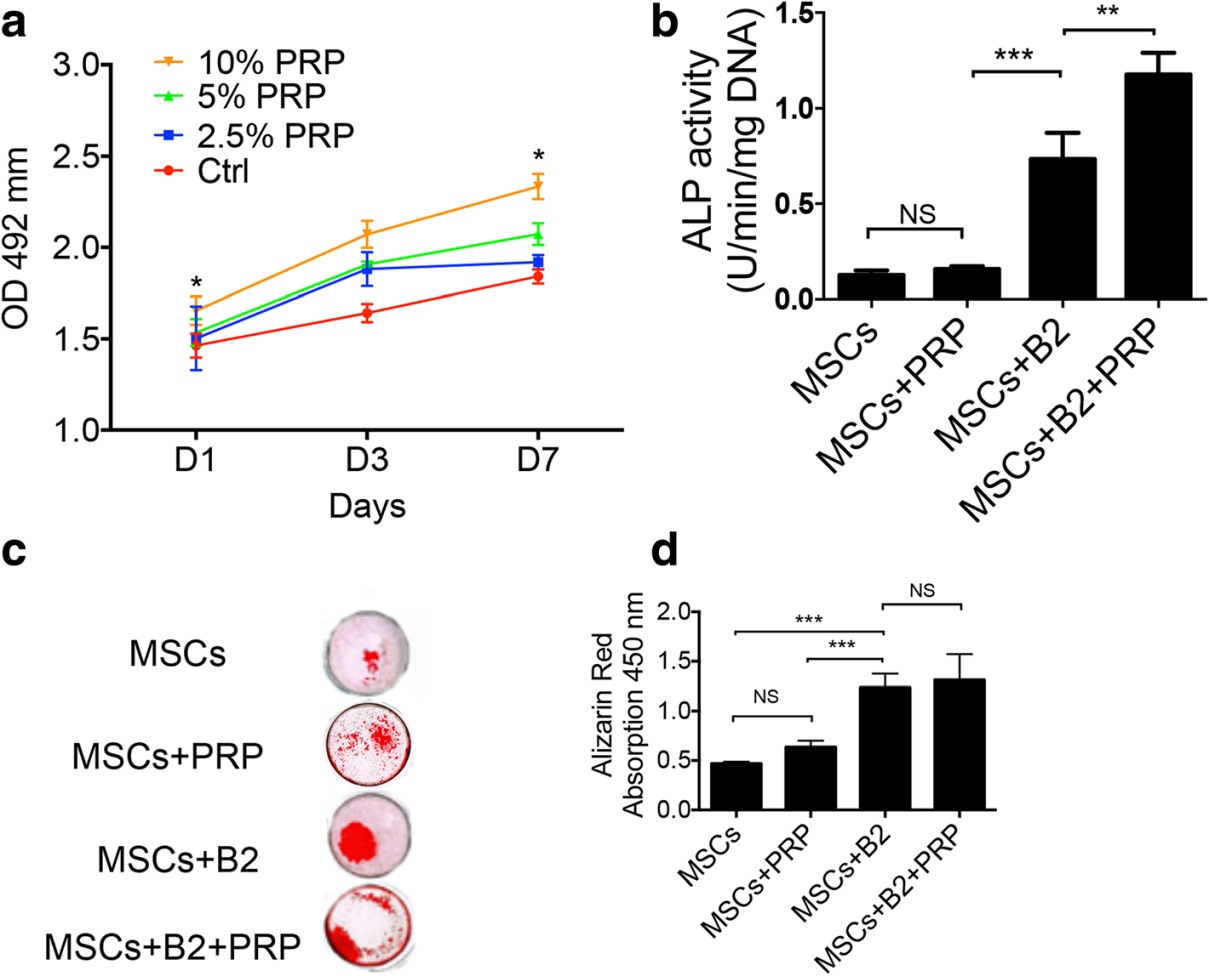
PRP promotes proliferation and osteogenic differentiation of BMP2-modified MSCs in vitro. **a** Proliferation of MSCs with different PRP concentrations (n = 4). The cell seeding density is 5 × 10^4^ per well in a 96-well plate. MSCs + PRP (10%) group has significantly higher cell proliferation rate at days 1, 3, and 7 as compared with other groups and is indicated by ^*^ *p* <0.05. **b** ALP activity of MSCs, MSCs + BMP2 (MSCs + B2), MSCs + PRP, and MSCs + B2 + PRP. The cells were induced with osteogenic media for 7 days. Data represent the mean ± SD of  3 samples. The ALP activity of MSCs + B2 + PRP group was significantly higher than the other groups as marked as ^**^ *p* < 0.01; ^***^ *p* < 0.0001; *NS:* not statistically significant. **c** Alizarin Red staining of MSCs, MSCs + B2, MSCs + PRP, and MSCs + B2 + PRP. Cells were cultured in osteogenic medium for 21 days. **d** Quantitative analysis (n = 5) of cell mineralization from Alizarin Red staining shown in C. ^***^ *p* < 0.0001; *NS:* not statistically significant. *ALP* alkaline phosphatase, *B2* bone morphogenetic protein 2, *MSCs* mesenchymal stem cells, *nCS* nano-calcium sulfate, *PRP* platelet-rich plasma

PRP has been reported to promote cell proliferation, therefore, we first examined whether PRP could also promote BMP2-modified MSCs proliferation. All groups showed an increase over the 7-day study. At day 7, there was a general increase in cell number with increasing concentration of PRP. The group of 10% PRP concentration had significantly higher cell proliferation than all other concentration groups (Fig. 1a).

**Fig. 2:**
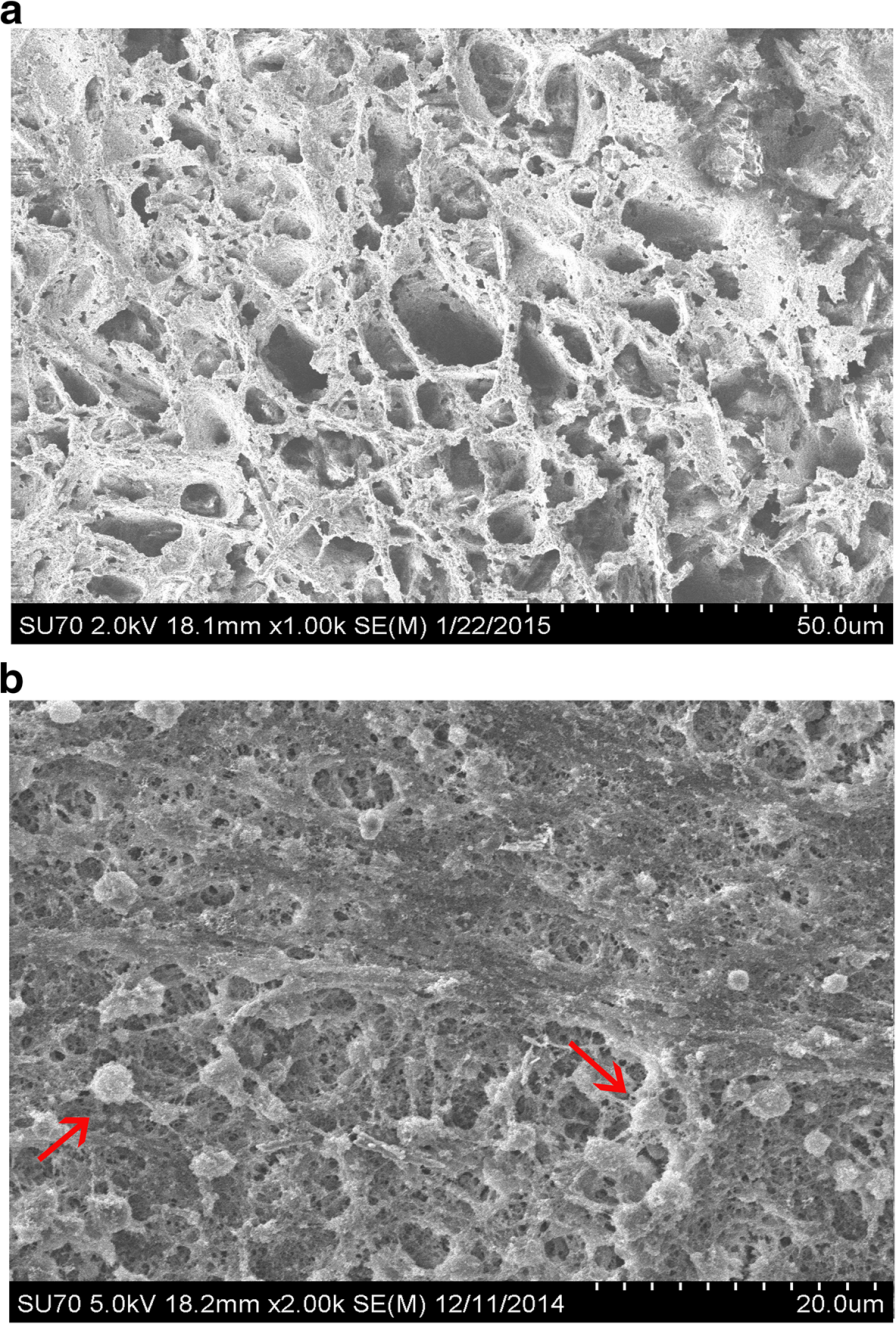
Scanning electron microscopic (SEM) view of nCS discs (**a**) and nCS/10% PRP scaffold (**b**). *Red arrows*: platelets

The ALP activity and the level of calcium deposition are important consideration factors for evaluating osteoblast differentiation. MSCs with BMP2 and/or PRP were induced with osteogenic medium for 7 days and then were subjected for ALP activity assay. As expected, BMP2 modification (MSCs + B2) dramatically increased ALP activity (*p* < 0.0001, Fig. 1b). However, adding 10% PRP to BMP2-modified MSCs (MSCs + B2 + PRP) could further enhance ALP activity (*p* < 0.01). The level of calcium mineral deposition after 3 weeks in culture was investigated by Alizarin Red staining (Fig. 1c). The quantitative results showed that calcium deposition in the groups of MSCs + B2 + PRP and MSCs + B2 were significantly higher than that in the other groups (*p* < 0.0001) (Fig. 1d). Adding PRP to BMP2-modified MSCs slightly but not significantly promotes calcium deposition (*p* < 0.05). Furthermore, we studied the structure of nCS and nCS/PRP gel. SEM analysis showed that nCS scaffolds are polyporous with an average pore diameter of 70–80 μm (Fig. 2a) and the platelets could attach on the nCS/PRP scaffolds (Fig. 2b).

**Fig. 3:**
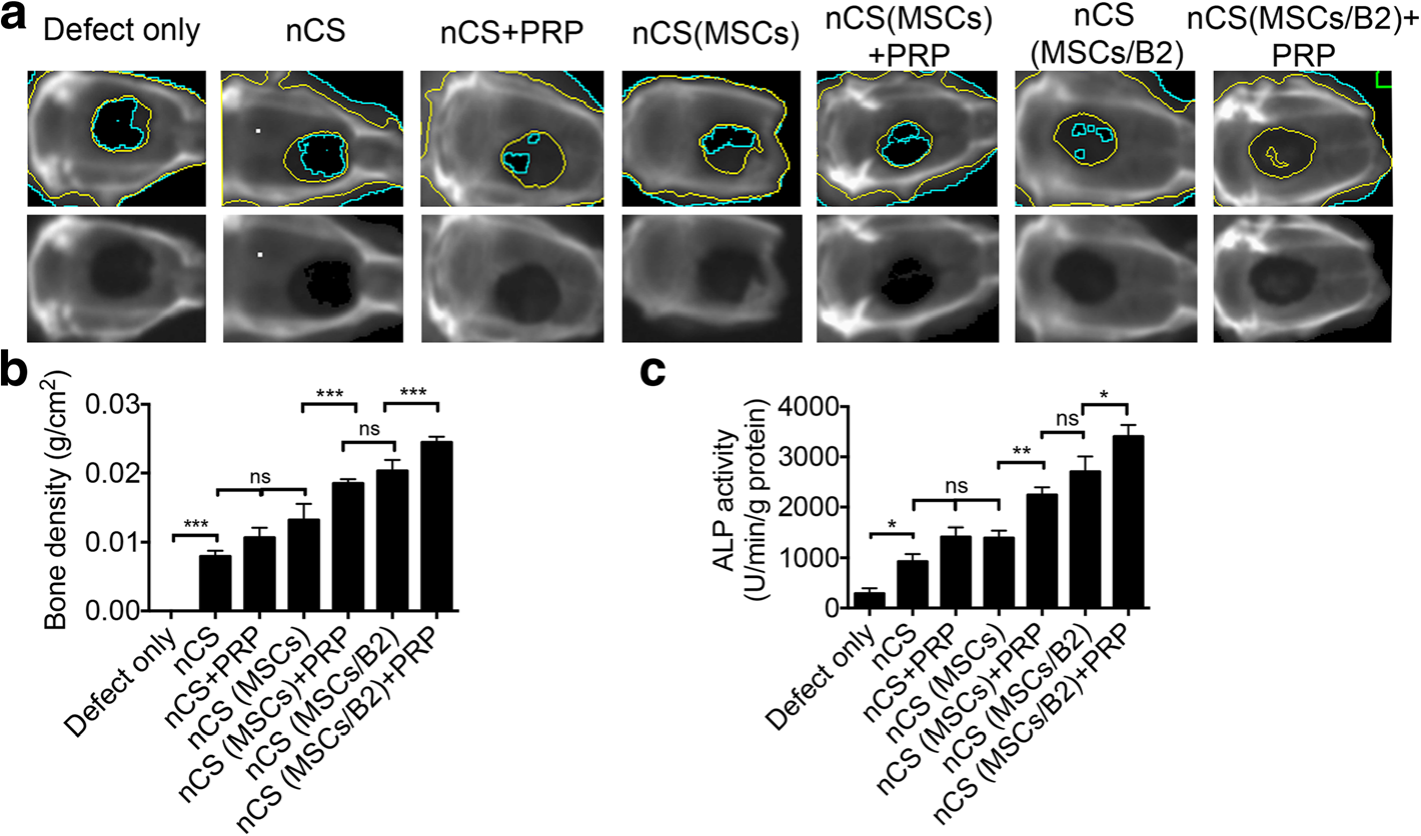
Bone mineral density analysis. **a** Images of calvarial bone obtained using LUNAR PIXImus system, 8 weeks after surgery. General X-ray view (*lower row*) and X-ray with color outlined bone regeneration images (*upper row*) are showed. The *black area circled* with *green line* indicates low density area (fibrous tissue). The *gray area* between the *green line* and the *yellow lines* is the newly-formed bone area. **b** Quantitative analysis of bone density from A (n = 3). **c** ALP activity of newly formed bone (n = 3). ^*^ *p* < 0.05; ^**^ *p* < 0.01; ^***^ *p* < 0.001; *ns:* not statistically significant. *ALP* alkaline phosphatase, *B2* bone morphogenetic protein 2, *MSCs* mesenchymal stem cells, *nCS* nano-calcium sulfate, *PRP* platelet-rich plasma

nCS/PRP gel delivery system with BMP2 gene-modified MSCs promotes bone regeneration in vivo. To evaluate the potential of the nCS/PRP delivery system and BMP2 gene-modified MSCs for bone in vivo, 8-mm bone defects were created in the calvarial bones of 8-week-old SD rats and calvarial bones were harvested at 8 weeks post-surgery. We first examined the bone formation with a BMD test (bone mineral density). The result showed that the nCS (MSCs/B2) + PRP group exhibited greater bone density than the other groups (Fig. 3a and b), confirming that the combination of nCS (MSCs/B2) with PRP significantly promotes bone regeneration. The nCS (MSCs/B2) group showed less bone density than nCS (MSCs/B2) + PRP, however, the bone density is comparable between nCS (MSCs/B2) and nCS (MSCs) + PRP groups, suggesting PRP is as powerful as BMP2 in promoting bone regeneration (Fig. 3a and b). Similar bone density was shown in the groups of nCS, nCS + PRP, and nCS (MSCs), but those were all higher than the defect-only group (Fig. 3a and b). To further test the bone regeneration activity in different groups, we tested the ALP activity of freshly harvested samples. Consistently, nCS (MSCs/B2), nCS (MSCs) + PRP and nCS (MSCs/B2) + PRP groups showed higher ALP activity compared with other groups, implying more robust osteogenic activity (Fig. 3c). The nCS (MSCs/B2) + PRP group had the highest ALP activity (Fig. 3c), demonstrating that PRP promotes osteogenic differentiation.

**Fig. 4:**
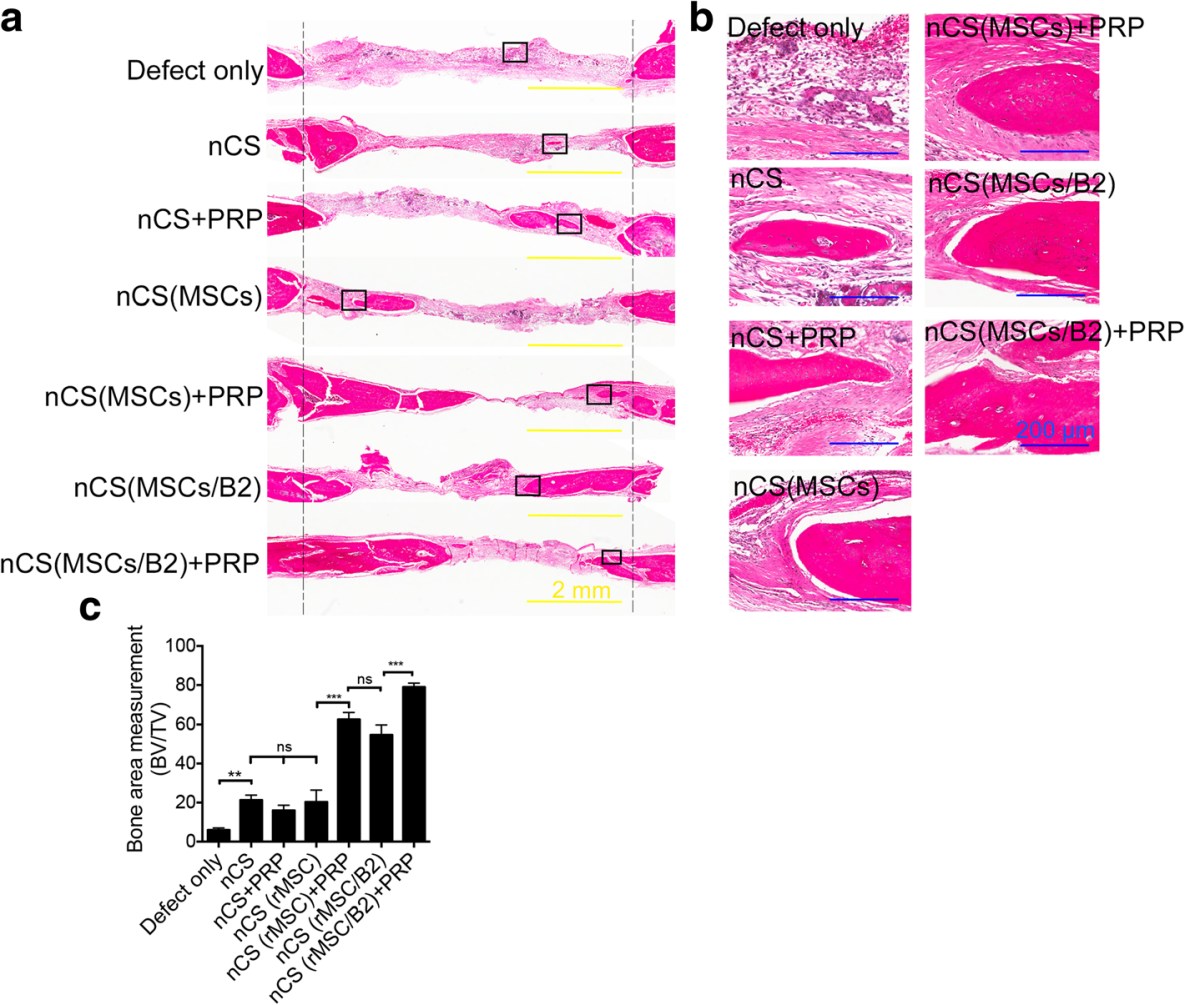
Histological analysis of newly formed bone. **a** Hematoxylin and eosin staining of coronal sections through the midline of defects (between *dashed line*). Combination of nCS/PRP scaffold containing BMP2-modified MSCs promotes new bone formation. Scale bar =  2 mm. **b** Higher magnification of hematoxylin and eosin staining shown in A (*black rectangle*). Scale bar  =  200 μm. **c** Quantitative analysis of newly formed bone (BV) to the total implant area (TV) (n = 3). ^**^ *p* < 0.01; ^***^ *p* < 0.001; *ns:* not statistically significant. *B2* bone morphogenetic protein 2, *MSCs* mesenchymal stem cells, *nCS* nano-calcium sulfate, *PRP* platelet-rich plasma

Hematoxylin and eosin-stained sections showed that at 8 weeks following the implantation, no residual materials or inflammatory infiltrating cells were seen within any of the defect regions (Fig. 4a and b). In all treatment groups, the smallest amount of new bone was formed in the defect-only group compared with other groups, and most of defect areas were covered by fibrous-like tissues (Fig. 4a and b). The groups of nCS, nCS + PRP, and nCS (MSCs) formed small pieces of new bone, however showed no significant difference, and most of the defect areas were also covered by fibrous-like tissues (Fig. 4a and b). The nCS (MSCs/B2) + PRP group had larger bone formation in the bone defect area compared with nCS (MSCs) + PRP and nCS (MSCs/B2) groups (Fig. 4a and b), suggesting both BMP2-modified MSCs and PRP are critical for bone formation. Histomorphometric analysis showed that the amount of newly formed bone (BV) to the total implant area (TV) in the nCS (MSCs/B2) + PRP group was significantly greater than those in the other six groups (Fig. 4c).

**Fig. 5:**
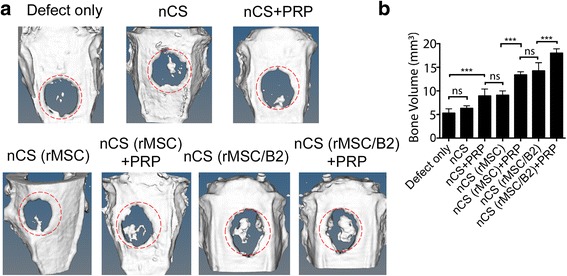
Micro-CT analysis of newly formed bone: **a** Images of calvarial bone obtained using micro-CT, 8 weeks after surgery. Higher amount of bone was detected in the nCS (rMSC/B2) + PRP group as compared to other groups. **b** Quantitative analysis of bone volume in the implanted area from A. The average bone volume were analyzed (n = 4). ^**^ *p* < 0.01; ^***^ *p* < 0.001; *ns:* not statistically significant.* B2* bone morphogenetic protein 2, *MSCs* mesenchymal stem cells, *nCS* nano-calcium sulfate, *PRP* platelet-rich plasma

Furthermore, we tested the bone regeneration in the defects by micro-CT. As expected, those critical-sized bone defects cannot heal by themselves, therefore, the defect-only group (no treatment) and the scaffold-only groups (nCS) showed less bone regeneration in the defects compared with the groups with MSCs or PRP (Fig. 5a). Moreover, nCS (MSCs/B2) and nCS (MSCs) + PRP have similar new bone formation, suggesting PRP and BMP2 are all important for new bone formation (Fig. 5a). However, PRP combined with MSCs/BMP2 in the nCS (MSCs/B2) + PRP group significantly enhanced bone formation, comparing with nCS (MSCs/B2) or nCS (MSCs) + PRP, demonstrating the function of the combination of PRP and BMP2-modified MSCs in inducing bone regeneration (Fig. 5a and b). Quantitative bone volumes in the defect areas analysis demonstrated significantly higher values in nCS (MSCs/B2) + PRP group as compared with the other groups (*p* < 0.05), exhibiting robust osteogenic activity (Fig. 5b).

## Discussion

For improving healing of critical-sized calvarial bone defects, until now, the major concern has been the poor integration of biomaterial scaffold and cells with the neighboring tissue, which slows down bone regeneration. This study, for the first time, investigates the effect of the combination of BMP2-modified MSCs with nCS and PRP fibrin gel scaffolds on healing critical-sized bone defects. Our results demonstrated the efficiency of this system in bone regeneration, and emphasized the potential ability to employ this system for bone defect treatment.

Calcium sulfate has good biocompatibility, osteoconductivity, and degradation rate for bone regeneration [25, 26]. As an extension of the study by He et al. [15], we used nCS along with 10% PRP fibrin gel as a delivery system to increase the surface area for stem cells attachment and to provide growth factors for promoting cell differentiation and bone regeneration. Platelets consist of many activated growth factors that could advance bone regeneration. However, it gets exhausted within 24 hours, if delivered directly to the site, which prevents it from performing its role in regeneration [20, 27]. Hence, a proper delivery system is essential for applying platelets. Our results showed that the combination of PRP and MSCs significantly increased the osteogenic differentiation and proliferation, which can be reasonably explained since PRP consists primarily of PDGF, IGF and TGF-beta, which promote osteoblast proliferation and maturation [27]. Moreover, PRP can be derived from whole blood and is biocompatible to the cells.

In this study, by transplanting BMP2-modified MSCs with nCS and PRP fibrin gel into well-established critical-sized rat calvarial bone defects, we found that the BMD of this group was much higher than that in other groups and exhibited dramatic bone repair in bone defects at 8 weeks following the surgery. Histological analysis complemented the BMD result and all defects were almost covered with newly- mineralized bone tissue. These results demonstrated that the combination of BMP2-modified MSCs with an nCS/PRP gel scaffold is an effective approach for bone regeneration and repair. Notably, the critical-sized defects were repaired much better in the MSCs-containing groups compared with the group without MSCs, and exhibited no sign of rejection in all groups, indicating that most of the implanted allogeneic MSCs maintained their viability and had no apparent immunorejection by the host. This conclusion was supported by some previous findings. For example, Li et al. [28] found that there is no rejection and/or graft versus host disease by intravenously injecting green fluorescent protein-labeled allogeneic MSCs into rabbits. Saito et al. [29] demonstrated that the rats display immunological tolerance to mouse MSCs.

Recombinant BMP2 has to be present in high concentrations (up to milligrams) in order to induce an osteogenic effect, and clinical applications are limited to a single dose at the time of implantation. While the use of lentivirus and retrovirus vector has disadvantages including gene integration into genomic DNA, which could lead to the mutation, and life-time and uncontrolled ectopic expression of the target genes, likely causing tumor or other severe side effects. Therefore, we used a recombinant adenoviral vector carrying human *BMP2* gene (Adv-BMP2) to transduce MSCs in order to prolong the BMP2 activity [30]. Our previous study showed that adenovirus-mediated BMP2 expression lasts up to 14 days, which is sufficient to stimulate bone regeneration and repair [15]. Here, besides the results that support the concept that BMP2-engineered MSCs enhance bone regeneration, the effects of PRP and BMP2 delivery in MSCs observed in this study highlights the importance of BMP2, PRP, and MSCs in bone healing. When PRP is used in conjunction with MSCs in vitro, the findings demonstrated that it is inclined toward promoting the cellular proliferation of MSCs [31]. Although PRP also consists of a small amount of BMP2, however, the increased proliferative activity may lead to altered morphology of the cell distribution as well as poor differentiation [32, 33]. BMP2 stimulates the differentiation and maturation of MSCs [34]. When ectopic BMP2 was used in conjunction with PRP, we observed that PRP could synergistically promote the activity of BMP2 thus increasing the proliferation activity and the osteogenic potential of MSCs [32, 33].

## Conclusions

In summary, this study provides the first evidence to support that this novel delivery system of nCS/PRP sandwich-like scaffold combined with MSCs/B2 strongly stimulates bone formation. This system can be useful not only in critical-sized bone defects, but also in defects with limited accessibility, such as in repairing periodontal bone defects. Its use in minimally invasive techniques, such as in situ fracture fixation and percutaneous vertebroplasty to fill the lesions and strengthen osteoporotic bone, is another area to pursue further.

## Additional file

Additional file 1: Figure S1. PRP and scaffold preparation. (A) PRP preparation by two times centrifuge. *PRP* platelet-rich plasma, *PPP* platelet-poor plasma. (B) Schematic of nCS/PRP scaffold preparation, and MSCs loading. *nCS* nano-calcium sulfate, *B2* BMP2. (C) Representative images of nCS disks before and after PRP loading. 8-mm defect was created in rat calvaria bone, and scaffolds were put into the defects. (TIF 2456 kb)

## Abbreviations

ALP: Alkaline phosphatase
BMPs: Bone morphogenetic proteins
GFs: Growth factors
IGF: Insulin-like growth factor
MSCs: Mesenchymal stem cells
nCS: Nano-calcium sulfate
PDGF: Platelet-derived growth factor
PPP: platelet-poor plasma
PRP: Platelet-rich plasma
TGF-beta: Transforming growth factors beta
