# Supplementary figures and images for: The combination of nano-calcium sulfate/platelet rich plasma gel scaffold with BMP2 gene-modified mesenchymal stem cells promotes bone regeneration in rat critical-sized calvarial defects
Source: Stem Cell Res Ther. 2017 May 25;8:122. doi: 10.1186/s13287-017-0574-6 (PMC5445399; doi:10.1186/s13287-017-0574-6)

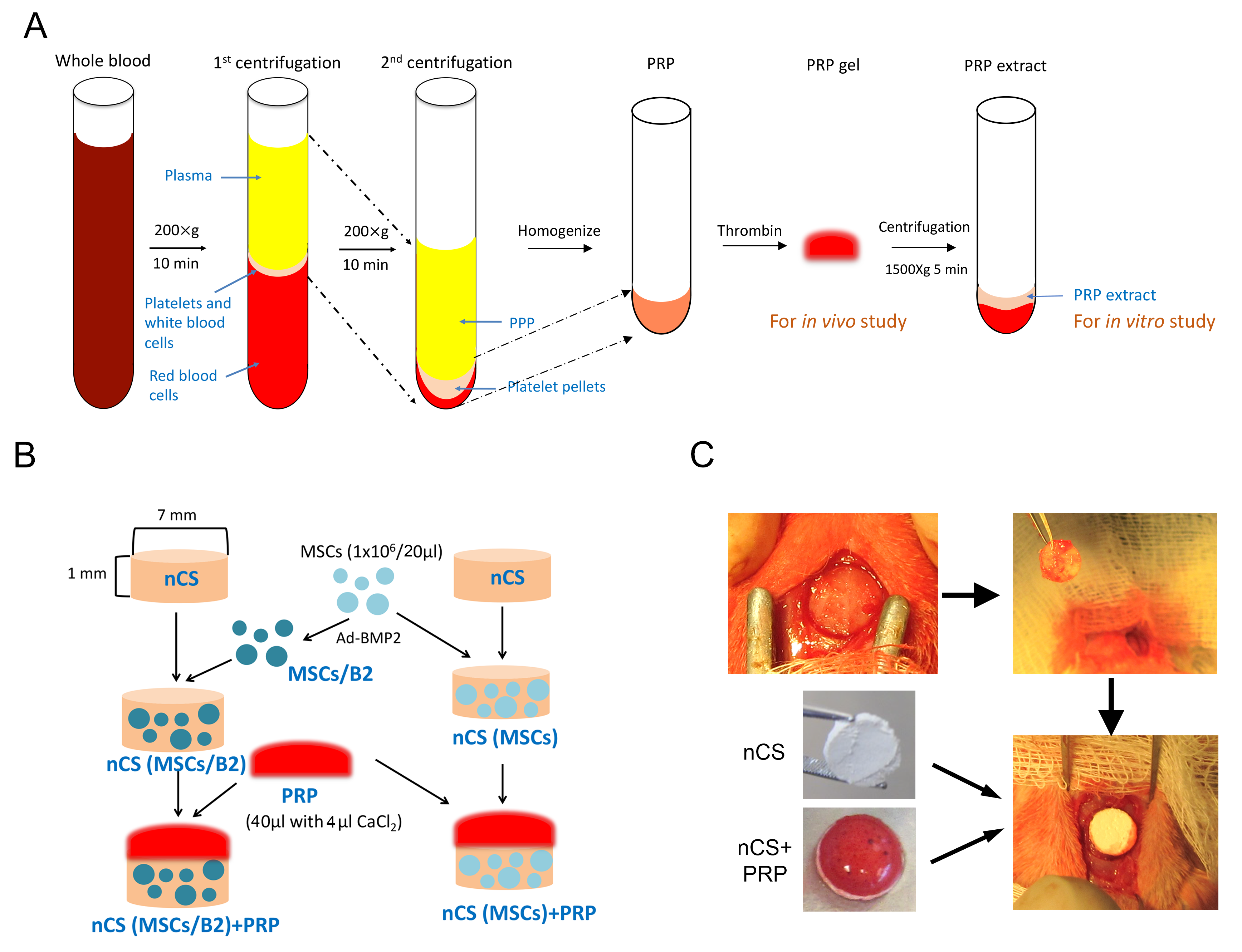

Supplement: Additional file 1: Figure S1. — PRP and scaffold preparation. (A) PRP preparation by two times centrifuge. PRP platelet-rich plasma, PPP platelet-poor plasma. (B) Schematic of nCS/PRP scaffold preparation, and MSCs loading. nCS nano-calcium sulfate, B2 BMP2. (C) Representative images of nCS disks before and after PRP loading. 8-mm defect was created in rat calvaria bone, and scaffolds were put into the defects. (TIF 2456 kb) [file 13287_2017_574_MOESM1_ESM.tif]
